# The Effect of Migratory Beekeeping on the Infestation Rate of Parasites in Honey Bee (*Apis mellifera*) Colonies and on Their Genetic Variability

**DOI:** 10.3390/microorganisms9010022

**Published:** 2020-12-23

**Authors:** Laura Jara, Carlos Ruiz, Raquel Martín-Hernández, Irene Muñoz, Mariano Higes, José Serrano, Pilar De la Rúa

**Affiliations:** 1 Departamento de Zoología y Antropología Física, Facultad de Veterinaria, Universidad de Murcia, Campus de Espinardo, 30100 Murcia, Spain; jaran.laura@gmail.com (L.J.); cruizcar@ull.edu.es (C.R.); irenemg@um.es (I.M.); jserrano@um.es (J.S.); 2 Departamento de Biología Animal, Edafología y Geología, Universidad de La Laguna, 38206 La Laguna, Spain; 3 IRIAF, Instituto Regional de Investigación y Desarrollo Agroalimentario y Forestal, Laboratorio de Patología Apícola, Centro de Investigación Apícola y Agroambiental (CIAPA), Consejería de Agricultura de la Junta de Comunidades de Castilla-La Mancha, 19180 Marchamalo, Spain; rmhernandez@jccm.es (R.M.-H.); mhiges@jccm.es (M.H.); 4 Instituto de Recursos Humanos para la Ciencia y la Tecnología (INCRECYT, ESF), Fundación Parque Científico y Tecnológico de Albacete, 02006 Albacete, Spain

**Keywords:** managed colonies, parasitic infestation rate, *Varroa destructor*, *Nosema* spp., deformed wing virus, genetic diversity

## Abstract

Migratory beekeeping is a widely extended practice aimed at increasing the yield of products and pollination services of honey bee colonies. However, it represents a stress factor, as it facilitates the dissemination of diseases and may compromise the genetic identity of the colonies involved. To analyze the extent of these effects, pathogens infestation rate and genetic composition were monitored in a field experiment comparing stationary and migratory colonies sharing the same environmental conditions but differing in management (stationary vs. migratory) and genetic background. We studied the pathogens infestation rate (*Varroa destructor*, *Nosema* spp., and Deformed Wing Virus (DWV)) at four different times: before migratory operation, two weeks later, at the end of the migratory period, and two weeks after the return of the migratory hives. An increased incidence of *V. destructor* and *Nosema ceranae* and a lower DWV viral load were found in migratory colonies. Temporary changes in genetic diversity were detected regardless of colony type, suggesting that stressors other than management affect the genetic diversity of the colonies. Our study demonstrates that migratory practices have variable effects on the health and genetic diversity of honey bee colonies, which should be taken into account for the development of sustainable beekeeping.

## 1. Introduction

The importance of the honey bee (*Apis mellifera*) is related to its crucial role in the pollination of wild plant species and in agroecosystems [1,2,3]. The economic benefits of beekeeping are derived both from the products generated, including honey, wax, pollen, and royal jelly, and the pollination that honey bees provide to many crops [4]. This pollination activity is even more important in the case of migratory beekeeping, as crops and wild plants from different regions and different flowering seasons benefit from honey bee foraging behavior [5].

There has been a demise of honey bee colonies in North America and Europe over the last few decades, with beekeepers reporting unexpected high winter losses of about 30% among managed honey bees in the last decade [6,7,8,9]. To date, large-scale surveys of managed honey bee populations in the US and Europe have been unable to identify a single factor responsible for this colony loss. This has led researchers to hypothesize that a combination of factors is acting in synergy to compromise honey bee survival [10,11,12,13,14]. Among the most important factors that have a negative impact on the longevity of honey bee colonies are parasites, primarily the mite *V. destructor* [15,16] and its associated viruses [17,18,19] as the Deformed Wing Virus (DWV) [20], and pathogens such as the microsporidia from the genus *Nosema*, specifically *Nosema ceranae* [21,22,23,24] and several other bacterial and fungal brood pathogens [25,26,27]. Other factors believed to enhance the detrimental effects of these parasites and pathogens are pesticide exposure [28], poor nutrition [29], reduced genetic diversity [30], and queen failure [31], as well as beekeeper expertise [32] and current honey bee management practices such as migratory beekeeping [33].

To date, the potentially harmful effects of migratory beekeeping have seldom been investigated [34,35,36,37]. Honey bee confinement, vibration and noise, and marked changes in colony temperature are among the negative influences on colonies associated with such practices. Moreover, the installation of colonies in new pollination locations might increase their exposure to pesticides [34,36,38] and pathogens [39,40]. These colonies must also adapt to new environmental conditions and potential stressors, including orientation cues, daily oscillations in temperature, humidity, and wind regimes. A study of the impact of migratory management on honey bee health and their stress response showed a significant decrease in lifespan and an increase in oxidative stress in migratory as opposed to stationary worker honey bees in the USA [38]. Migratory practices also lead to higher drifting rates [41], which increases the horizontal transmission of pathogens and parasites [42]. In fact, transportation and pollination services were recently proposed to increase the infestation rate and abundance of *Nosema ceranae* [39] and some viruses [33,43] in *A. mellifera* worker bees.

In Spain, migratory beekeeping is a common practice due to the marked seasonality of the Mediterranean climate. About 80% of the 2.8 million of colonies in Spain are moved in an annual cycle (data from 2017 [44]) that involves transportation over distances of at least 400 km in summer, from the southern and warmer regions of the Iberian peninsula to the northern regions with a milder climate and later flowering season. The extended practice of migratory beekeeping in Spain represents an evident risk for disease spread and a higher infestation rate of pathogens. However, this practice favors the gene flow between migratory and stationary colonies over a large geographic scale in the Iberian Peninsula [45,46]. At the intra-colony level, the environmental conditions of the different settlements of apiaries and the proliferation of pathogens may also affect the genetic diversity in colonies, as both are important selective factors [12,47,48]. Furthermore, there is accumulating evidence that genetic variation can influence host susceptibility to pathogens [49,50,51]. As a result, we set out to determine the effects of migratory beekeeping on the infestation rate of important pathogens (*V. destructor*, *Nosema* spp. and DWV) in honey bee colonies in Spain. The hypothesis tested was that stress factors associated with migratory management affect colony health and disease transmission, resulting in a higher infestation rate of pathogens and viruses in the migratory colonies. We also aimed to determine whether migratory beekeeping has an effect on the genetic diversity in the individual colonies, comparing the changes in genetic diversity between migratory and stationary colonies from the same apiary. It is hypothesized that factors associated with migratory beekeeping favor particular patrilines within the colonies, depending on their selective value against external stressors prevailing in the migratory areas. Additionally, changes in the genetic composition of colonies due to queen replacement and its subsequent mating in the region of migration are also expected, which is a practice that represents a significant mechanism of gene flow among honey bee colonies over large geographic distances in Spain.

## 2. Materials and Methods

### 2.1. Ethical Statement

The study was conducted under the supervision of researchers of the University of Murcia and the Regional Institute for Food and Forestry Research and Development, Spain. Beekeeping practices were performed in compliance with the Spanish Ministry of Agriculture, Fisheries and Food regulations.

No permits were required to conduct the study.

### 2.2. Experimental Design

**Figure 1:**
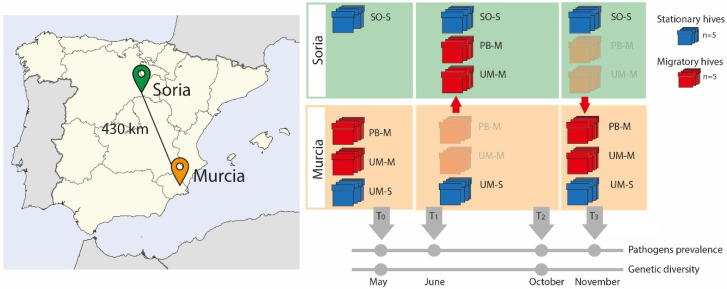
Graphic representation of the sample design. The map on the left shows the location of the two localities from where the hives were moved (Murcia) to the new location (Soria). On the right are representations of the two types of hives sampled (red = migratory, blue = stationary) and the timeline indicating the months in which samples were taken for the different analyses. (UM means Murcia, PB is professional beekeeper, SO is Soria, M = migratory, S = stationary).

Following a similar research study [35] in this field experiment, ten sentinel hives from the apiary located at the Campus of the University of Murcia (Southeast Spain) were divided into two groups of five hives each. While one group remained stationary (UM-S) during the experiment, the second was moved (UM-M) 430 km to the region of Soria (Northern–Central Spain) during the summer (June–October 2015) (Figure 1). This latter group was moved together with another five hives belonging to a professional beekeeper from Murcia (PB-M) who usually carries out migratory beekeeping. The transported colonies were settled in Soria around 13 km from a stationary apiary of a hobbyist beekeeper (SO-S), thereby sharing the same environmental conditions. Five of these stationary colonies were also sampled. At the beginning of October, the UM-M and PB-M colonies were transported back from Soria to Murcia in the same truck. Thus, samples investigated in the experiment came from four groups of colonies, namely, two from the university campus of Murcia (hives that remained in Murcia, UM-S and those transported to Soria, UM-M), one from the professional beekeeper (hives transported to Soria, PB-M), and that of stationary colonies from Soria (SO-S).

This experimental design was established to assess the effects of migratory movements in a single year, as environmental factors are not homogeneous from one year to the following. In this design, we included groups of migratory (UM-M and PB-M) and stationary (UM-S and SO-S) hives. Comparisons were made between (i) groups sharing similar genetic background but subjected to different management strategies (UM-M vs. UM-S); (ii) groups with different genetic backgrounds but subjected to the same management (UM-M vs. PB-M); and (iii) groups settled in the same location (Soria) sharing the same environmental conditions but with different genetic background (UM-M vs. PB-M), and different management strategies (SO-S) (Figure 1). See Table S1 for colony codes.

### 2.3. Sampling Dates and Collection

The 20 colonies included in the experiment were sampled on four different months in 2015: in May before moving the hives from Murcia to Soria (T_0_); in June, two weeks after transportation and settling the migratory hives in Soria (T_1_); in October, at the end of the migratory season (T_2_); and in November, two weeks after transportation of hives back to Murcia (T_3_). The stationary colonies from Soria (SO-S) were only sampled during the migratory season (T_1_ and T_2_), when the UM-M and PB-M hives were in their proximity (Figure 1). Samples of adult honey bee workers were taken from combs located between those with sealed brood and those with fresh honey in order to sample both nursing and foraging workers [52]. Samples were kept either in absolute ethanol at −20 °C or frozen directly at −80 °C. Due to colony die-off, we were unable to sample honey bees from all 20 colonies at each time point, as explained later.

### 2.4. Varroa Destructor Detection

The infestation rate of *V. destructor* was estimated from about 200 worker honey bees per colony at each sampling time. A total of 70 samples were evaluated: 15 colonies (5 UM-M, 5 PB-M and 5 UM-S) and 4 sampling times, plus 5 colonies of the SO-S apiary sampled at T_1_ and T_2_. Varroa mites were dislodged from honey bee workers preserved in ethanol after being shaken, and the proportion of infested individuals per colony was calculated by dividing the number of mites counted by the number of honey bees in each sample [53].

### 2.5. Extraction and Purification of DNA and RNA

Simultaneous RNA and DNA extraction was carried out to detect *Nosema* spp. and to quantify DWV. For this, the abdomen of 22 honey bees from each colony and sampling time were macerated in AL buffer [54]. The abdomen of each single honey bee was removed and placed in one well of a 96-well plate containing glass beads (2 mm; Sigma^®^, St. Louis, MO, USA) and then macerated in 180 µL of AL buffer (Qiagen^®^ 19075, Hilden, Germany) and shaken in a Tissuelyser (Qiagen^®^) for 6 min at 30 Hz. Afterward, the homogenate was transferred to another plate (Deepwell, Eppendorf^®^, Hamburg, Germany) and incubated for 10 min with 20 µL of proteinase K (10 mg/mL) and 200 µL of the AL buffer at 70 °C, shaking at 300 rpm.

DNA and RNA purification was performed simultaneously in a Biosprint 96 workstation (Quiagen^®^, Hilden, Germany) following the BS96 DNA Tissue extraction protocol. Total nucleic acids (RNA and DNA) were eluted in 100 µL of elution buffer, and aliquots of 75 µL of this eluted product were frozen until further processing to detect *Nosema* spp. The remaining 25 µL obtained from each honey bee from the same colony and sampling time were pooled (hereafter referred to as pooled cDNA) [55], and any genomic DNA was completely removed by digestion with DNase I (Quiagen^®^ kit 79254). Ten µL of this DNA-free pooled RNA were used to generate cDNA using the iScriptTM cDNA Synthesis Kit (Biorad^®^, Hercules, CA, USA). Negative and positive controls were run in parallel for each step (maceration, nucleic acid extraction, and reverse transcription).

### 2.6. Nosema spp. Detection

Aliquots of 2.5 µL of the total nucleic acids eluted from the abdomen of each individual honey bee were used to detect *N. apis* and *N. ceranae*. PCR reactions were performed as described previously [56], using an internal PCR control to determine the reliability of the analysis. The infestation rate per colony was estimated from the number of nosema positive honey bee individuals divided by the total number (22 per colony) of honey bees analyzed [57].

### 2.7. Quantification of DWV

Quantification of the viral load of each colony and time was performed by qPCR in a 384-well plate using the LightCycler^®^ 480 Real Time PCR System (Roche^®^, Basel, Switzerland). The qPCR was carried out in a total volume of 10 µL containing 2× LightCycler^®^ 480 Probes Master, 0.3 µM of the primers DWV958F and DWV9711R, 0.1 µM of the FAM marked probe DWV9627T [58], and 2.5 µL of the pooled cDNA. The qPCR conditions were 95 °C for 10 min, and 45 cycles of 95 °C 10 s and 60 °C 40 s (annealing temperature). Each sample was run in duplicate, and the average Cq values were calculated. Positive individuals were only considered when the average Cq value was <40 [59]. A known amount of DWV PCR product was run in parallel to obtain a standard curve that was used to convert the Cq data to the initial concentration of DWV RNA in the reaction tube. Positive and negative PCR controls were also included. The online tool “dsDNA copy number calculator” (hosted in http://cels.uri.edu/gsc/cndna.html) was utilized to calculate the equivalence between amount (ng) of DWV RNA and DWV copy number in each qPCR reaction. Viral load was calculated for each colony and sampling time by averaging the amount of RNA extracted from 80.5 mg of honey bee abdomens. To calculate the viral load per individual honey bee, we determined the average amount of RNA in a single honey bee by taking into account the elution volume and the average weight of one honey bee [55]. Prior to data analysis, virus copy number was log10 transformed to account for the exponential distribution of the data [60]. Since zero values cannot be log-transformed, negative samples were assigned a hypothetical Cq value of 40 and later converted to the theoretical virus copy number as described above.

### 2.8. Number of Combs

The number of combs with a capped brood per colony was annotated for every colony at each of the four sampling times, as a qualitative measure of their strength and general health status [61]. This also served to assess the extent to which this characteristic, often evaluated by beekeepers when checking health and vitality status of the colonies, is related to pathogen infestation rate.

### 2.9. Statistical Analysis

Calculation and graphical representation of the different descriptive measures were implemented with the R v. 3.2.2 software [62]. The normal distribution of the data obtained of each group of colonies and the equality of variance between the groups of data were tested with the Kolmogorov–Smirnov normality test and the F-test of equality of variances, respectively. Thereafter, T-tests or Mann–Whitney-U tests if required were used to compare means between groups. All these analyses were carried out in PAST v.3.14 [62]. In collapsing colonies, the number of brood combs was considered as zero, and the pathogen infestation rate and viral load were considered as not available (NA) at the sampling times subsequent to colony collapse. Possible correlations between the different variables were assessed with the Pearson’s correlation test using PAST v.3.14.

A Principal Component Analysis (PCA) was also performed with PAST v.3.14 software to compare the relative weight of the different variables (brood combs, pathogens infestation rate, and virus load), as well as to assess the explanatory power of these variables on the dispersion of the colonies, according to the type of beekeeping management.

### 2.10. Sampling for Genetic Diversity and Patrilineal Composition of the Colonies

Honey bee workers were sampled at T_0_ (May) prior to the movement of the five migratory UM-M hives to Soria, and at T_2_ (October) after the migratory season (Figure 1). The analysis of the genetic diversity of 48 workers per colony that results from the queen and the multiple droned she mated with, allows the assessment of the impact of transhumance due to queen replacement and subsequent mating with local drones. Samples of 48 worker honey bees per colony and sampling time were preserved in absolute ethanol at −20 °C until processed.

### 2.11. Microsatellite Amplification and Detection

The determination of the number of patrilines and the genetic diversity were inferred from the analysis of five microsatellite loci [63]. The total DNA from each worker honey bee (48 per colony) was extracted using the Chelex^®^ method [64], and 2 µL of this DNA was used for multiplex microsatellite amplification in a total volume of 10 µL containing 1× reaction buffer, 1.2 mM MgCl_2_, 0.3 mM of each dNTP, 0.4 µM of each primer (one of each pair fluorescence labeled), and 1.5 units of Taq polymerase (Biotools^®^, Madrid, Spain). The annealing temperature was set at 54 °C, and the PCR products were visualized by capillary electrophoresis (ABI-3730, Applied Biosystems^®^, Foster City, CA, USA), scoring individual alleles using GeneMapper v3.7 software (Applied Biosystems^®^).

### 2.12. Microsatellite Data Analysis

The following population genetic parameters were calculated for each group of colonies (migratory and stationary), and at each sampling time (T_0_ and T_2_) based on allele frequency and using the GenAlex v. 6.41 software [65]: expected heterozygosity (He), number of different alleles (Na), number of effective alleles (Ne), and number of private alleles (Pa). Fisher’s exact test was applied to detect genetic differentiation [66] in colonies between T_0_ and T_2_ in Genepop On the Web (http://genepop.curtin.edu.au/genepop_op3.html). Honey bee worker genotypes were analyzed with the Colony 2.0.5 software [67] to unravel the patrilineal composition of each colony at each sampling time. Events of queen replacement and honey bee drifting were also extrapolated with this software.

## 3. Results

### 3.1. Parasite and Pathogen Assays

#### 3.1.1. Varroa Destructor

**Figure 2:**
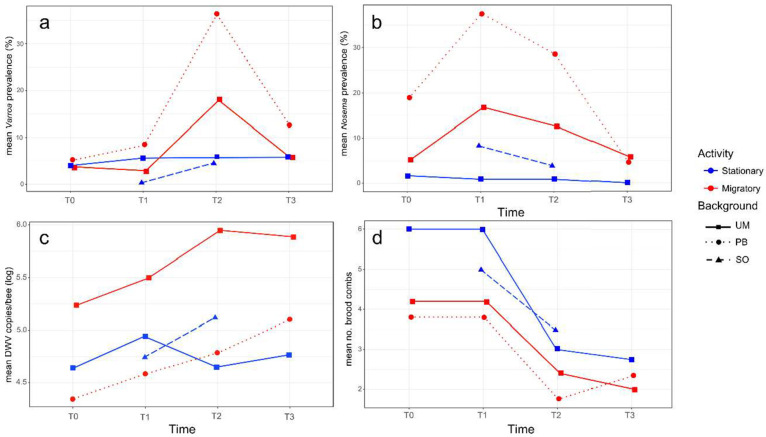
Average data per colony group (stationary in blue and migratory in red), genetic background (squares for the University of Murcia (UM), circles for the professional beekeeper (PB) and triangles for Soria (SO)) and sampling time (T_0_, T_1_, T_2_ and T_3_) of the variables: (**a**) infestation rate of *V. destructor*; (**b**) infestation rate of *N. ceranae*; (**c**) log10 DWV copy number/bee; (**d**) number of brood combs.

A low infestation rate of *V. destructor* was detected in all the colonies at the beginning of the study (T_0_: mean values 4.5 ± 6.2% in the migratory that is UM-M plus PB-M groups, and 4.0 ± 2.3% in the stationary colonies, that is UM-S plus SO-S). No significant changes (N = 35, *p* > 0.05) were observed after transportation to Soria in the migratory and stationary colonies respectively (T_1_: 5.7 ± 11.1% and 3.0 ± 4.0%), although a significant increase (N = 39, z = −2.165, *p* = 0.030) in the infestation rate of varroa was detected in migratory colonies after the summer period (T_2_: 26.1 ± 29.2%); the infestation rate in stationary colonies remained low (T_2_: 5.2 ± 6.8%). Indeed, the former displayed a significantly higher mite infestation rate than the stationary colonies at T_2_ (N = 19, t = 2.205, *p* = 0.042) (see Figure 2a for mean values per group), with colonies 1M (UM-M), 7M (PB-M), and 10M (PB-M) registering the highest infestation rate at T_2_ (42%, 83%, and 61% respectively, Table S1).

When comparing migratory and stationary colonies from the University of Murcia (same genetic background), a trend toward a higher infestation rate of varroa mites (N = 10, t = 1.503, *p* = 0.078) was observed in the migratory colonies at T_2_ (UM-M: 18.0 ± 14.2% vs. UM-S: 8.0 ± 9.1%). This difference was not significant (N = 8, *p* > 0.05) after the colonies returned to Murcia at T_3_, due to a decrease in *V. destructor* infestation rate in these migratory group of colonies (UM-M: 8.56 ± 11.04%), while the infestation rate remained stable in the stationary colonies (UM-S: 5.8 ± 3.9%). Migratory colonies with different genetic backgrounds, UM-M and PB-M, displayed a distinct response to *V. destructor*, as the PB-M colonies suffered a stronger increase in varroa infestation rate (from T_0_: 5.12 ± 8.57% to T_2_: 36.28 ± 41.98%) than the UM-M colonies (from T_0_: 3.78 ± 2.28% to T_2_: 17.96 ± 9.06%) in the migratory period. The standard deviation was also higher in the PB-M hives, showing large differences in varroa infestation rate among individual colonies within the group (Table S1). The stationary colonies in Soria (SO-S) that shared the location and environmental conditions with UM-M and PB-M colonies from June to October (T_1_ and T_2_) showed the lowest infestation rate of *V. destructor* in June (T_1_: 0.28 ± 0.63%). This rate increased slightly in October (T_2_: 4.58 ± 4.59%), showing a similar value to that recorded in the UM-S group at that time (T_2_: 5.76 ± 9.06%).

#### 3.1.2. *Nosema* spp.

The total number of worker honey bee individuals analyzed by PCR was 1413, as four colonies died off throughout the experiment (Table S1). *Nosema* spp. were detected in 10.9% of them; concretely, *N. ceranae* was detected in 84% of the infected workers, *N. apis* was detected in 8% and co-infection with both species was detected in the remaining 8%. Co-infection increased from T_0_ to T_1_ (Table S1), but since the *N. apis* infestation and co-infection rates were low and unsuitable for proper statistical analysis, the following data mainly refer to *N. ceranae* infection.

The infection rate of the microsporidium was distinct among the colonies at the beginning of the experiment possibly due to the random selection of the hives analyzed (Figure 2b), as at T_0_, the infection rate of *N. ceranae* in migratory colonies was 9.9 ± 10.03% and 1.7 ± 1.7% in the stationary colonies. At T_1_, the infection rate of *N. ceranae* increased significantly (N = 35, z = −2.387, *p* = 0.017) in migratory (T_1_: 26.8 ± 15.74%) but not in the stationary hives, as UM-S colonies maintained a lower infection rate (T_1_: 0.9 ± 2.0%). Therefore, a higher *N. ceranae* infection rate was detected in migratory as opposed to stationary colonies at T_1_ (N = 20, z = −3.184, *p* = 0.002). After the summer period (T_2_), the infection rate of this microsporidium tended to decrease in the two groups of colonies, yet the migratory colonies (T_2_: 18.1 ± 15.54%) still maintained a higher infection rate of *N. ceranae* (N = 19, z = −2.892, *p* = 0.004) than the stationary colonies (T_2_: 2.1 ± 3.15%). Two weeks after their transportation back to Murcia (T_3_), the infection rate of *N. ceranae* kept the decreasing trend in the migratory colonies.

Between the two groups of migratory colonies (with different genetic background), PB-M colonies had a greater infection rate of *N. ceranae* (T_1_: 36.7 ± 14.57%) than UM-M colonies after they were transported to Soria (T_1_: 16.8 ± 9.9%; N = 10, z = −2.089, *p* = 0.037).

#### 3.1.3. DWV Loads

From the 65 pools of RNA extracted from 22 worker honey bees per colony and at the distinct sampling times, DWV was detected in 35 pools (53.9%) with mean viral loads ranging from 2.22 × 10^4^ to 6.89 × 10^6^ DWV copies/bee (Table S1). Due to the random selection of the hives, the initial viral load at T_0_ differed widely among the colonies irrespective of their management. However, at T_1_, the average DWV load tended to increase for the migratory and stationary colonies, albeit not significantly (N = 35, *p* > 0.05). After the migratory period, at T_2_, there were no clear trends in DWV load between the stationary and migratory colonies (Figure 2c). In general, the largest differences were detected among the individual colonies over the entire study period (T_0_ to T_3_), although a temporal fluctuation at the colony level was also observed, irrespective of the management group.

The UM-M and UM-S colonies registered a distinct initial situation, with a tendency (although not significant, N = 10, t = 1.80, *p* = 0.31) toward higher DWV loads in UM-M (T_0_: 8.13 × 10^5^ ± 1.38 × 10^6^ DWV copies/bee) than in UM-S (T_0_: 6.58 × 10^4^ ± 7.14 × 10^4^ DWV copies/bee). In June (T_1_), both groups of colonies showed a slight increase in DWV copies/bee, while at T_2_ (at the end of the migration period), the DWV load had increased in UM-M (T_2_: 1.4 × 10^6^ ± 1.5 × 10^6^ copies/bee), whereas it had decreased in UM-S (T_2_: 4.4 × 10^4^ ± 6.0 × 10^6^ copies/bee). Nevertheless, no significant differences (N = 10, t = 2.03, *p* = 0.07)) were found in the DWV load between the two groups at T_2_ due to the high standard deviation registered. Likewise, similar viral loads were found in the two groups of colonies at T_3_ (Figure 2c).

For migratory colonies that differed in their genetic background (UM-M and PB-M), the initial situation was also distinct. The DWV viral load was below the detection threshold in all the PB-M colonies at T_0_, while the UM-M colonies registered an average viral load of 4.45 × 10^4^ ± 2.47 × 10^4^ DWV copies/bee. However, despite the initial differences, both groups showed a tendency toward a higher DWV load at T_1_ (UM-M: 6.7 × 10^5^ ± 9.6 × 10^5^ and PB-M: 2.4 × 10^4^ ± 1.5 × 10^4^ copies/bee) and T_2_ (UM-M: 1.4 × 10^6^ ± 1.5 × 10^6^ and PB-M: 1.8 × 10^5^ ± 3.4 × 10^5^ copies/bee), while at T_3_, the DWV load tended to decrease slightly in UM-M (8.1 × 10^5^ ± 6.3 × 10^5^ copies/bee) and tended to increase (although not significantly, N = 9, t = 0.36, *p* = 0.73) in PB-M (7.6 × 10^5^ ± 1.3 × 10^6^ copies/bee). In the case of the SO-S colonies established close to the migratory area, a tendency toward an increased DWV load was observed from T_1_ (8.2 × 10^4^ ± 1.4 × 10^5^ copies/bee) to T_2_ (7.9 × 10^5^ ± 1.7 × 10^6^ copies/bee, *p* > 0.05), while in the colonies settled in Murcia (UM-S), a tendency toward a decrease in DWV load was registered in the same period (T_1_: 1.1 × 10^5^ ± 1.36 × 10^5^ to T_2_: 4.4 × 10^4^ ± 6.0 × 10^6^ copies/bee, *p* > 0.05).

#### 3.1.4. Brood Combs

There was a slightly higher mean number of brood combs in stationary as opposed to migratory colonies at the beginning of the experiment (T_0_: 5.5 ± 2.1 and 4.0 ± 3.0, respectively). The same number of brood combs was also found at T_1_, two weeks after the transportation of the migratory colonies to Soria. At T_2_, the colony 6M (from the PB-M group) collapsed, and fewer brood combs were detected after the summer period in both groups of colonies (T_2_: 3.2 ± 1.6 and 2.1 ± 1.0 respectively, Figure 2d). This decrease was significant in the stationary group (N = 10, t = 2.680, *p* = 0.016), although at T_3_, after transporting the migratory hives back to Murcia, a similar low mean number of brood combs was observed in the migratory and stationary colonies (2.14 ± 0.7 and 2.8 ± 1.9, respectively). Colonies 1M (UM-M), 4S (UM-S), 6M, and 7M (PB-M) had collapsed by that time (T_3_).

#### 3.1.5. Correlation Analysis

**Figure 3:**
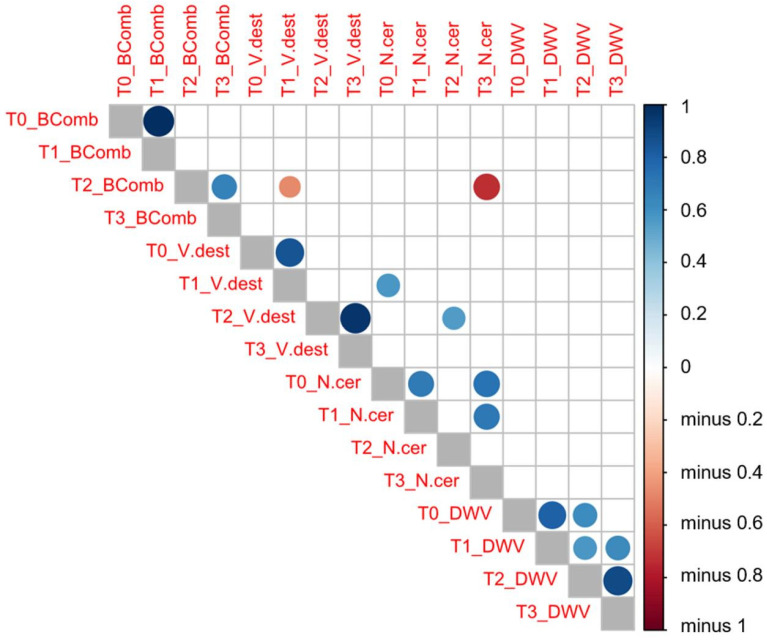
Pearson’s correlation tests between the variables analyzed (*V. destructor* (V.dest) and *N. ceranae* (N.cer) infestation rate values, DWV load (DWV) and number of brood combs (BComb)) at each sampling time (T_0_, T_1_, T_2_, and T_3_). Dark blue means a positive correlation between these two variables, whereas dark red means a negative correlation as indicated in the vertical bar on the right side of the figure.

A Pearson’s correlation test (Figure 3) between the variables analyzed and the sampling times highlighted a positive correlation in the number of brood combs between T_0_ and T_1_ (same number of brood combs, r = 1, *p* = 0.000) and also between T_2_ and T_3_ (r = 0.662, *p* = 0.001). However, no correlation was found between the number of brood combs recorded in T_1_ and that observed in T_2_ (*p* > 0.05).

An increased infestation rate of *V. destructor* at T_1_ was negatively correlated (r = −0.471, *p* = 0.042) with the number of brood combs at the next sampling time (T_2_). This negative correlation was stronger in migratory (r = −0.672, *p* = 0.033) than in stationary colonies (r = −0.472, *p* = 0.042). Similarly, this negative correlation was also found in migratory colonies (r = −0.691, *p* = 0.039), whereby the infestation rate of *V. destructor* at T_2_ was negatively related to the mean number of brood combs at T_3_. Likewise, the lower number of brood combs at T_2_ appeared to be strongly correlated to the higher infection rate of *N. ceranae* a few weeks later (T_3_: r = −0.731, *p* = 0.011), although there was no correlation between the number of brood combs and the DWV loads. The infection rate of *N. ceranae* at T_0_ appeared to be positively correlated to the *V. destructor* infestation rate at T_1_ (r = 0.571, *p* = 0.026) and T_2_ (r = 0.551, *p* = 0.014), yet there was no correlation between varroa or nosema infestation rate and DWV load.

#### 3.1.6. PCA Analysis

**Figure 4:**
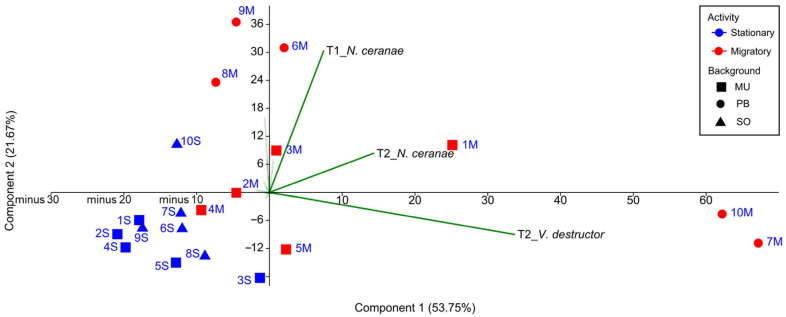
Scatterplot of the colonies showing dissimilar dispersion of stationary and migratory colonies according to the rates of parasitism. The code of the colonies has been abbreviated for better visualization (stationary in blue and migratory in red, squares for the University of Murcia (UM), circles for the professional beekeeper (PB) and triangles for Soria (SO)).

A first PCA analysis, including all the colonies and taking as variables the number of brood combs, varroa and nosema infestation rate, and DWV viral load at the four sampling times (Table S1), allowed us to assess the relative weight of each variable in the clustering of the colonies throughout the study (T_0_–T_3_), without making any prior assumption regarding beekeeping management. Eigenvalues of the two principal components explained 75.42% of the overall variability. The infestation rate of *V. destructor* and *N. ceranae* at T_2_ (PC 1) and the infection rate of *N. ceranae* at T_1_ (PC 2) were the variables with the strongest influence on the dispersion of the colonies in the PCA (Figure 4). The dispersion of the migratory colonies was based on the most prevalent pathogens (UM-M-3, PB-M-8, PB-M-6, and PB-M-9 along the T_1__*N. ceranae* vector, UM-M-1 along with T_2__*N. ceranae*, and UM-M-5, PB-M-7, and PB-M-10 along with T_2__*V. destructor* vector), while the dispersion of the stationary colonies was not affected by these vectors. A second PCA with only the data from UM-M and UM-S colonies (the same genetic background) showed a similar result with same variables influencing the dispersion, i.e., *V. destructor* and *N. ceranae* at T_2_, and the infection rate of *N. ceranae* at T_1_.

### 3.2. Genetic Diversity and Patrilineal Composition of the Colonies

#### 3.2.1. Genetic Diversity

In order to assess the impact of beekeeping management on the genetic variation of the colonies, population genetic parameters were inferred from the genotypes of 48 worker honey bees per colony before (T_0_) and after migration (T_2_). Colonies in Soria (SO-S) were sampled only in T_1_ and T_2_, and both datasets were also included in this analysis. In total, 1728 worker honey bees were genotyped, since one colony died off in T_2_. Temporal variation of the population parameters was not statistically significant in the migratory or stationary colonies, although differences were observed in the mean number of alleles, in the number of private alleles, and in the genetic diversity in some individual colonies (Table 1). In general, the greatest differences in genetic diversity were found among individual colonies (e.g., UM-M-5 vs. PB-M-7 or UM-S-2 vs. SO-S-7), irrespective of their management (migratory and stationary) or sampling time (T_0_ and T_2_). To further analyze the temporal differences between T_0_ and T_2_, Fisher’s exact test was performed for each colony. Significant genetic differences (*p* < 0.001) were detected in seven colonies (UM-M-1, UM-M-3, UM-M-4, PB-M-7, PB-M-8, PB-M-9, and PB-M-10) within the group of migratory colonies, whereas in the stationary colonies, significant differences (*p* < 0.001) were only found in the UM-S-4 and SO-S-8 colonies.

#### 3.2.2. Patriline Analysis, Queen Replacement, and Drifting Workers

Paternal lines within the colonies and the relationships among the worker offspring were studied at T_0_ and T_2_. The number of patrilines ranged from 7 to 15 in the migratory and from 5 to 18 in the stationary colonies. Temporal variation in the number and frequency of patrilines was observed in individual colonies from both groups (Table 2). Despite the equivalent number of patrilines in colonies UM-M-1, UM-M-2, UM-S-1, UM-S-2, and UM-S-4, a replacement of some patrilines was detected between T_0_ and T_2_, with some low frequency patrilines no longer detected and new patrilines recorded. At the colony level, some patrilines were more frequently found at one particular sampling time. Despite the trends observed in favor of some patrilines rather than others in both individual migratory and stationary colonies, no selective patterns could be detected related to the management.

No replacement of the honey bee queen was detected in the stationary colonies but in six of the migratory colonies: UM-M-1, UM-M-3, UM-M-4, PB-M-7, PB-M-8, and PB-M-10. This replacement was seemingly related to the genetic differences at the beginning of the experiment and those observed between T_0_ and T_2_. In the colonies UM-M-1, UM-M-3, PB-M-8, and PB-M-10, worker offspring from the new queen were only detected at T_2_, suggesting that the replacement of the queen occurred in Soria during the migratory period. For colony UM-M-4, worker offspring from the new queen were already present at T_0_, which indicates that the replacement and mating of the new queen took place in Murcia prior to transportation of the colonies to Soria. For colony PB-M-7, worker offspring from three different queens were detected, which indicates that the replacement and mating occurred in both periods (T_0_ and T_2_). Drifting workers (Table 2) were detected in 16 colonies at T_0_ (nine migratory and seven stationary) and in 10 colonies (five migratory and five stationary) at T_2_, although these values were not significantly different.

## 4. Discussion

This study provides further insights into the impact of migratory beekeeping practices on honey bee health [35,36] and on the infestation rate of pathogens and diseases in the colonies [39,40,68]. Our major finding is that migratory beekeeping and its associated stress lead to higher *V. destructor* and *N. ceranae* infestation rates in the colonies, which partially confirms our initial hypothesis.

In the case of *V. destructor*, the type of colony management (migratory vs. stationary) seems to influence the proliferation of the mite during the summer months. Although the two groups of migratory and stationary colonies display similar low varroa infestation rates in May (T_0_) and June (T_1_), a significantly higher infestation rate of *V. destructor* was recorded in migratory colonies compared to the stationary colonies after the summer period (T_2_). *Varroa destructor* population dynamics are known to be highly influenced by the host population dynamics [16]. Indeed, the increase of mites in migratory colonies may be partially explained by the existence of sufficient brood combs [69,70], thanks to favorable weather conditions in Soria and the availability of ample resources. However, colonies that are permanently located in Soria did not show such a large increase in varroa mite, despite sharing the same environmental conditions. Thus, the stress associated with migratory management can also contribute to a high infestation rate of the pathogen. For example, phoretic infestation by mites from highly infected colonies to healthy ones during migratory operations have been noted [41]: a clear case of horizontal pathogen transmission [42]. Genetic background is another factor to be considered in terms of the susceptibility of colonies to pathogens [46,71], which may explain the higher increase in *V. destructor* infestation rate after summer (T_2_) in PB colonies (owned by the professional beekeeper) compared to displaced UM colonies. It should be noted that both PB and UM colonies were transported together, placed in the same site in Soria, and showed similar varroa infestation rates (after being submitted to the same varroa treatment) at the beginning of the experiment.

*Nosema ceranae* was more prevalent than *N. apis*, which is in consonance with previous reports in Spain [46,56,72] and elsewhere [21,22,73]. Despite the low rates of *N. apis* infection, the infection rate of combined infections of the two microsporidia species was higher in migratory colonies. This result is worth noting, as it has been demonstrated that combined nosema infection significantly decreases honey bee survival when compared to single species infection [74]. *Nosema ceranae* was significantly more prevalent in migratory than in stationary colonies in June (T_1_), just two weeks after the hives were transported to Soria. Its infection rate was also high in October (T_2_). Hence, management would be related to the infection rate of *N. ceranae* in *A. mellifera* workers [39]. It has been hypothesized that transportation to ensure pollination weakens the immune system of honey bees, making them more susceptible to infection. Stressful factors involved in this management that affect the honey bee immune system and that thereby favor pathogen proliferation, including truck vibration, noises, marked temperature changes during transport of the hives, and the release of nosema spores from infected honey bees that die during transportation [21,75,76,77], are events that inevitably occurred while moving the hives in this experiment. In addition to individual immunity, transhumance can affect social immunity. The resilience of a honey bee colony to a chronic stress factor, such as *N. ceranae*, is also based on its ability to maintain a homogeneous population, with the right balance between nurse and foragers workers. When this balance is altered, the compensation mechanisms of the honey bee colony can fail, thus weakening the colony, and yielding to a final collapse [78]. Transhumance can lead to a further loss of foragers honey bees, which unbalances the population and favors the spread of nosema.

The significant increase in *Nosema* spp. from T_0_ to T_2_ was not found at T_3_, after the migratory colonies had returned to Murcia (beginning of November). Factors that may have counterbalanced the negative effects of transportation are perhaps related to the better weather encountered by the honey bees on their return to their original location. During their stay in Soria, the temperatures were often close to 10 °C and rainfall was frequent (e.g., in mid-October, in the two weeks before their return, there was 40% of rainy days and a mean temperature of 11.2 °C). Cold temperatures and honey bee confinement are thought to enhance the infestation rate and density of *Nosema* spp. inside colonies through trophallaxis and oral–fecal routes [42,79,80]. When hives returned to Murcia, honey bees encountered only 10% of rainy days and a mean temperature of 18.5 °C, which are weather conditions that favor a lower infestation rate of microsporidia. Another explanation is the “natural dilution” that occurs in the number of infected honey bees due to the natural growth cycle of the colonies [23]. If the number of “new” honey bees increases faster than the transmission of the parasite within the colony, the relative percentage of infected honey bees decreases over time. This occurs most effectively in colonies with young queens and in periods of climatic and resource bonanza.

In terms of DWV load, the virus was detected in 53% of the samples analyzed, which is a similar result as that reported in France for adult *A. m. mellifera* bees from March to November [17]. No clear trends were found between stationary and migratory colonies during our survey, and the largest differences were found among individual colonies rather than between colony groups. The viral loads detected were comparable to those reported in asymptomatic colonies in other studies employing a similar methodology [55]. Despite the evident differences in DWV load among colonies in May (T_0_), a tendency toward a greater viral load (although not significant) was detected in the two groups of colonies (migratory and stationary) in June (T_1_). The seasonal load variation of DWV is known to closely follow that of the varroa mite due to the role of this mite as a vector of DWV [19,81]. Thus, DWV load usually increases as the honey bee season progresses [16] and *V. destructor* parasitism augments, mainly in spring and early summer [15].

We found a significant reduction in the number of brood combs present in the colonies in October (T_2_), probably reflecting the increase of varroa infestation. Higher infestation rates of *V. destructor* at T_1_ were negatively correlated with the strength of the colony at T_2_ (inferred from the number of brood combs), both in migratory and stationary colonies. This result agrees with a number of studies where *V. destructor* is identified as the main factor affecting colony strength [59]. Indeed, we found that migratory colonies that collapsed before the end of the study had a high infestation rate of varroa mites. In addition, colonies with fewer brood combs at T_2_ also had higher levels of nosema a few weeks later (T_3_) after their return to Murcia. This observation may be related to the above mentioned: the colonies having less brood, had less non-infected young honey bees and therefore, there is a relative increase of parasitized honey bees. That is, the population of the parasite grows faster than the honey bee colony and thus increases the relative percentage of infected honey bees.

A positive correlation was detected between *V. destructor* and *N. ceranae* infestation rates, which is consistent with previous studies showing that weak colonies (less than five brood combs) are more frequently associated with higher numbers of pathogens in adult honey bees [81,82]. Unexpectedly, no correlation was found between *V. destructor* infestation rate and DWV load, in contrast to many studies that point to varroa as the most important vector for DWV, positively affecting the DWV copy number in honey bees [83]. However, some studies have shown a lag in the correlation [19] between the mite and the virus [84], suggesting that the epidemic’s dynamics are unique to individual colonies or even to different times during the season [85]. As summarized by the PCA analyses, the infection rate of *N. ceranae* in June (T_1_) and the infestation rate of *V. destructor* and *N. ceranae* in October (T_2_), appeared to be the variables that most strongly influence the status of migratory and stationary colonies.

In relation to the genetic diversity, migratory beekeeping had only a weak impact on the colonies. Short migration periods may be insufficient to produce changes on the overall genetic diversity, although trends were evident within the colonies in relation to allele frequency. This is exemplified by the fact that the strongest differences in genetic parameters were found between individual colonies irrespective of the management group or the sampling time (T_0_ or T_2_). Temporal changes in allele frequencies and the patriline composition of each colony were also greater within each management group than between groups, suggesting the influence of other non-management stressors affecting the colonies individually. Stressors such as the pathogen infestation rate and climatic condition are important selective forces that shape the genetic composition of colonies [12,47,48]. Furthermore, there is accumulating evidence that genetic variation can influence host susceptibility to pathogens [49,50,51]. Previous studies found different incidence of *N. ceranae* [86] and bacterial pathogens [87] among patrilines inside the colonies, as well as changes in allele frequencies and the signal of selection determined by varroa and nosema parasitism [46,88,89]. As climatic conditions were shared by all migratory colonies in this study, temporal genetic variation may rather be influenced by the infestation rate and the synergic effects of the particular combination of pathogens affecting each colony [90,91]. These stressors, along with the queen replacement events detected in some migratory colonies, may be the causes of the significant genetic differences detected. Queen replacement, as well as brood diseases, high varroa infestation rate, and DWV load have been proposed as the main factors leading to colony collapse in migratory beekeeping operations [31]. Moreover, queen replacement during migration and the subsequent mating of the queen in migratory areas, as observed in this study, have been also highlighted as an important factor for genetic homogenization and the loss of local adaptation in the Iberian honey bee populations [45,46]. However, the true impact of each individual stressor could not be weighed here due to the difficulty in controlling all the variables affecting the colonies in field conditions.

This study is a first attempt to identify the effects of migratory operations on honey bee colonies under field conditions, where the combined interactions of stress factors may have a significant effect on the colonies. Together, these findings indicate that genetic diversity is not notably affected by the type of beekeeping management. Thus, further studies are necessary to fully untangle these interactions between management, genetic background, environment, and other factors related to the status of the colony itself, weighing the effect of each factor on the honey bee colonies.

## Figures and Tables

**Table 1:** Population genetic parameters of migratory (M) and stationary colonies (S) at two sampling times: at the beginning of the experiment (T_0_, except the stationary colonies from Soria (SO-S) that were sampled at T_1_) and at the end of the migratory period (T_2_). Colony PB-M-6 ^†^ already died off at T_2_, therefore, data is not available (N.A.).

	He		Na		Ne		Pa	
Colony	T_0_	T_2_	T_0_	T_2_	T_0_	T_2_	T_0_	T_2_
UM-M-1	0.606	0.563	5.000	4.600	2.667	2.479	0.400	0.000
UM-M-2	0.610	0.594	4.400	4.000	2.644	2.505	0.200	0.000
UM-M-3	0.473	0.450	5.200	3.400	2.313	2.029	0.000	0.000
UM-M-4	0.598	0.531	5.800	4.200	2.992	2.378	0.200	0.200
UM-M-5	0.149	0.183	2.200	2.600	1.327	1.398	0.000	0.000
PB-M-6 ^†^	0.539	N.A.	4.200	N.A.	2.406	N.A.	0.000	N.A.
PB-M-7	0.636	0.627	6.200	6.600	3.115	2.925	0.000	0.000
PB-M-8	0.630	0.564	5.400	5.400	3.453	2.481	0.000	0.200
PB-M-9	0.507	0.501	4.600	4.000	2.498	2.389	0.200	0.200
PB-M-10	0.567	0.642	4.400	4.400	2.541	3.016	0.200	0.000
Migratory mean	0.532	0.517	4.740	4.356	2.596	2.400	0.120	0.067
UM-S-1	0.524	0.563	4.800	4.800	2.314	2.469	0.000	0.000
UM-S-2	0.375	0.360	4.200	3.400	2.040	1.980	0.000	0.000
UM-S-3	0.635	0.626	5.000	5.400	2.943	2.914	0.000	0.200
UM-S-4	0.561	0.565	5.600	4.200	2.554	2.558	0.000	0.000
UM-S-5	0.605	0.584	4.800	5.200	2.772	2.686	0.000	0.000
SO-S-6	0.638	0.582	5.800	4.200	2.816	2.441	0.200	0.000
SO-S-7	0.671	0.637	6.000	5.000	3.312	2.942	0.200	0.400
SO-S-8	0.557	0.619	6.800	6.000	3.100	2.842	0.400	0.400
SO-S-9	0.565	0.567	3.800	4.400	2.702	2.672	0.000	0.000
SO-S-10	0.421	0.486	4.000	4.600	2.020	2.189	0.000	0.000
Stationary mean	0.555	0.559	5.080	4.720	2.657	2.569	0.080	0.100
Total mean	0.543	0.539	4.910	4.547	2.626	2.489	0.100	0.084

(He = expected heterozygosity, Na = number of alleles, Ne = effective number of alleles and Pa = number of privative alleles).

**Table 2:** Number of patrilines, queen replacement events and drifting workers per colony and sampling times: at the beginning of the experiment (T_0_, except the stationary colonies from Soria (SO-S) that were sampled at T_1_) and at the end of the migratory period (T_2_). Colony PB-M-6 ^†^ already died off at T_2_; therefore, data are not available (N.A.).

	Number Patrilines		Queen Events		Drifting Workers	
Colony	T_0_	T_2_	T_0_	T_2_	T_0_	T_2_
UM-M-1	11	11		1		
UM-M-2	8	8			1	
UM-M-3	17	8		1	2	
UM-M-4 *	9 ^F1^/7 ^F2^	9 ^F2^	1		3	2
UM-M-5	7	6			1	1
PB-M-6 ^†^	15	N.A.	N.A.	N.A.	1	N.A.
PB-M-7 *	12 ^F1^/6 ^F2^	13 ^F3^	1	1	1	10
PB-M-8	8	14		1	2	
PB-M-9	7	8			1	1
PB-M-10	13	8		1	1	1
UM-S-1	13	13				1
UM-S-2	9	9			2	
UM-S-3	13	14				
UM-S-4	8	8			6	1
UM-S-5	16	18				
SO-S-6	11	9			3	
SO-S-7	15	16			6	
SO-S-8	15	17		1	11	3
SO-S-9	6	5			4	3
SO-S-10	10	11			2	1

Superscript F indicates the number of queens inferred from the worker genotypes. In the colonies labeled with an asterisk, it is shown the number of patrilines sired by each queen.

## Supplementary Materials

The following are available online at https://www.mdpi.com/2076-2607/9/1/22/s1, Table S1: Data obtained for each colony in the four sampling times (T_0_–T_3_).
