# Supplementary material for: The Effect of Migratory Beekeeping on the Infestation Rate of Parasites in Honey Bee (Apis mellifera) Colonies and on Their Genetic Variability
Source: Microorganisms. 2020 Dec 23;9(1):22. doi: 10.3390/microorganisms9010022 (PMC7822443; doi:10.3390/microorganisms9010022)
Supplement: Supplementary file 1 [file microorganisms-09-00022-s001.pdf]

The effect of migratory beekeeping on the infestation rate of parasites in honey bee (*Apis mellifera*) colonies and on their genetic variability

Laura Jara, Carlos Ruiz, Raquel Martín-Hernández, Irene Muñoz, Mariano Higes, José Serrano and Pilar De la Rúa

Table S1. Data obtained for each colony of the two groups: migratory (M) and stationary (S) in the four sampling times (T_0-T_3).

		V. destructor (%)				*N. ceranae* (%)				*N. apis* (%)				Co-infections by both *Nosema* spp (%)				DWV copies/bee				Brood combs (No.)			
**Code**	**Group**	**T_0**	**T_1**	**T_2**	**T_3**	**T_0**	**T_1**	**T_2**	**T_3**	**T_0**	**T_1**	**T_2**	**T_3**	**T_0**	**T_1**	**T_2**	**T_3**	**T_0**	**T_1**	**T_2**	**T_3**	**T_0**	**T_1**	**T_2**	**T_3**
**1UM**	**M**	2.6	1.4	42	NA	16.7	32	4	NA	0	0	0	NA	0	0	0	NA	2.22E+04	2.22E+04	2.15E+06	NA	0	0	3	0
**2UM**	**M**	5.7	4.6	10.8	5.8	0	15.8	8.7	4.5	0	0	0	0	0	0	0	0	1.11E+05	1.80E+05	3.28E+06	2.26E+06	8	8	2	2
**3UM**	**M**	1.2	5.6	7.8	3.4	9.1	13.6	32	4.5	0	0	0	9.1	0	4.5	0	0	3.22E+05	4.10E+06	5.13E+05	2.55E+06	6	6	2	2
**4UM**	**M**	8.7	2	9.4	4.4	0	13.6	0	0	0	0	9.1	0	0	0	0	0	3.56E+06	1.70E+06	6.89E+06	1.07E+06	3	3	3	3
**5UM**	**M**	0.7	1.1	19.8	8.5	0	4.5	9.1	4.8	0	0	0	0	0	0	0	0	5.14E+04	1.06E+05	2.22E+04	5.75E+04	4	4	2	1
**6PB**	**M**	20.3	36.7	NA	NA	28.6	31.8	NA	NA	0	0	NA	NA	0	4.5	NA	NA	2.22E+04	2.22E+04	NA	NA	6	6	NA	NA
**7PB**	**M**	2.2	4.8	82.7	NA	0.0	18.2	27.3	NA	0	0	0	NA	4.5	4.5	0	NA	2.22E+04	4.47E+04	2.22E+04	NA	4	4	0	NA
**8PB**	**M**	0.3	0.4	0.9	4.9	5.6	36.8	11.5	4.8	0	0	0	0	0	15.8	0	0	2.22E+04	2.22E+04	2.22E+04	2.22E+04	1	1	2	3
**9PB**	**M**	0	0	0.6	0	22.7	40.9	22.7	9.1	0	4.5	4.5	0	0	9.1	0	0	2.22E+04	4.32E+04	2.22E+04	2.22E+04	8	8	2	2
**10PB**	**M**	2.8	0	60.9	32.9	0.0	21.7	44	0	20	0	0	0	12	0	4	0	2.22E+04	9.00E+04	1.27E+06	4.14E+06	0	0	3	2
**1UM**	**S**	3.9	4.5	0	5.1	4.5	4.5	4.5	0	0	0	0	0	0	0	0	0	3.33E+04	5.53E+05	5.91E+04	2.22E+04	8	8	3	0
**2UM**	**S**	3	12.4	0	0.8	4	0	0	0	0	0	0	0	0	0	0	0	4.32E+04	2.22E+04	2.22E+04	2.22E+04	4	4	3	3
**3UM**	**S**	4.6	2	20.7	10.1	0	0	0	0	0	0	0	0	0	0	0	0	2.22E+04	2.22E+04	2.22E+04	2.22E+04	6	6	5	4
**4UM**	**S**	7.4	6.3	0	NA	0	0	0	NA	0	0	0	NA	0	0	0	NA	2.22E+04	4.84E+04	2.22E+04	NA	5	5	0	NA
**5UM**	**S**	1.2	3	8.1	7.2	0	0	0	0	0	0	0	0	0	0	0	0	2.08E+05	3.94E+05	2.74E+05	1.06E+06	7	7	4	4
**6SO**	**S**	NA	0	1.6	NA	NA	0	16	NA	NA	0	4	NA	NA	0	0	NA	NA	2.22E+04	2.22E+04	NA	NA	2	4	NA
**7SO**	**S**	NA	0	6.1	NA	NA	10	0	NA	NA	0	0	NA	NA	0	0	NA	NA	2.22E+04	2.46E+05	NA	NA	5	3	NA
**8SO**	**S**	NA	1.4	12	NA	NA	0	0	NA	NA	0	0	NA	NA	0	0	NA	NA	6.13E+05	6.89E+06	NA	NA	3	5	NA
**9SO**	**S**	NA	0	1.4	NA	NA	4.5	0	NA	NA	0	0	NA	NA	0	0	NA	NA	7.16E+04	4.66E+04	NA	NA	7	2	NA
**10SO**	**S**	NA	0	1.8	NA	NA	27.3	0	NA	NA	0	0	NA	NA	0	0	NA	NA	2.22E+04	2.22E+04	NA	NA	8	0	NA

NA means data not available because of colony death (those shaded in grey) or not sampled in a given time.
